# Exploring cotton SFR2’s conundrum in response to cold stress

**DOI:** 10.1080/15592324.2024.2362518

**Published:** 2024-06-05

**Authors:** Samantha M. Surber, Ngoc Pham Thien Thao, Cailin N. Smith, Zachery D. Shomo, Allison C. Barnes, Rebecca L. Roston

**Affiliations:** a Department of Biochemistry, University of Nebraska-Lincoln, Lincoln, NE, USA; b United States Department of Agriculture, North Carolina State University, Raleigh, NC, USA

**Keywords:** Abiotic stress, cold tolerance, arabidopsis, cotton, lipids, membranes

## Abstract

Cotton is an important agricultural crop to many regions across the globe but is sensitive to low-temperature exposure. The activity of the enzyme SENSITIVE TO FREEZING 2 (SFR2) improves cold tolerance of plants and produces trigalactosylsyldiacylglycerol (TGDG), but its role in cold sensitive plants, such as cotton remains unknown. Recently, it was reported that cotton SFR2 produced very little TGDG under normal and cold conditions. Here, we investigate cotton SFR2 activation and TGDG production. Using multiple approaches in the native system and transformation into *Arabidopsis thaliana*, as well as heterologous yeast expression, we provide evidence that cotton SFR2 activates differently than previously found among other plant species. We conclude with the hypothesis that SFR2 in cotton is not activated in a similar manner regarding acidification or freezing like Arabidopsis and that other regions of SFR2 protein are critical for activation of the enzyme than previously reported.

## Introduction

Cold temperature stressors are an increasing threat to crop production as the climate across the globe is becoming increasingly more unpredictable.^1,2^ The most concerning cold events for many crops are spring frosts during sensitive germination or early growth stages, and autumn hard freezes prior to harvest. These stressors can dramatically impact quality and yield, even crop loss. While many plants have evolved mechanisms to respond to and survive low temperatures, this crucial ability is often lacking in agricultural species re-domesticated to temperate regions (L.^3,4^ *Gossypium raimundii* (cotton) and *Zea mays* (corn) cultivars exemplify this vulnerability, as their response to cold is not fully understood.

Fortunately, much more is known about low-temperature tolerance in *Arabidopsis thaliana*. A naturally freezing tolerant plant, it acclimates to initial, above-zero chilling temperatures to enhance its below-zero freezing tolerance, especially through membrane remodeling.^5,6^ Membranes are a direct site of low-temperature damage, and tolerance requires membrane remodeling during both cold acclimation and additional low-temperature stress.^7–10^ In addition, soluble sugars and amino acids accumulate^11,12^ in response to a carefully controlled transcriptional and post-transcriptional set of cues.^13^

Specifically, SENSITIVE TO FREEZING2 (SFR2), a chloroplast enzyme classified as a glycosyl transferase, plays a pivotal role in Arabidopsis cold response. SFR2 is conserved in evolved land plants^14^ even in notoriously cold sensitive plants. SFR2 modifies the lipid monogalactosyldiacylglycerol (MGDG) by using it as a substrate and transfers the galactose headgroup to another MGDG producing DGDG (di-galactosyldicylglycerol). This process happens progressively to produce TGDG and TeGDG, respectively.^15,16^ This action is believed to stabilize membranes during freezing stress, and in Arabidopsis is completely dependent on the presence of SFR2.^15,17^ Notably, specific domains within Arabidopsis SFR2 beyond its core structure were identified as necessary for its activation and transferase activity. These include an unstructured loop region near the N-terminus and a portion of the C-terminus.^16^ Moreover, cytosolic acidification triggered by low temperatures has been established as a highly conserved step for SFR2 activation in Arabidopsis and other plant species.^7,18^

TGDG accumulation serves as a reliable proxy for SFR2 activity under cold or acid stress.^7,18^ A recent study comparing TGDG levels across diverse species described cotton, as a fascinating outlier, exhibiting minimal accumulation of TGDG under both normal and cold conditions, despite its close kinship to the highly accumulating model species, *Arabidopsis thaliana*. Cotton, a vital fiber and oilseed crop, has a myriad of varieties which results in many optimal growing temperatures for the genus.^19,20^ In any variety, it can be concluded that a rapid change in temperature whether heat or cold causes damage and yield loss for cotton.^21–26^ Most cotton is considered quite cold sensitive and it is grown in warmer regions of the world (National Cotton Council of America)^44^.

Because cotton is cold sensitive an unpredictable frost of 2007 decimated US crops, particularly in the cotton-rich Southeast, and it stands as a stark reminder of our vulnerability to climate instability.^27^ Because cotton is a major fiber and oilseed agricultural crop that responds differently than Arabidopsis to low temperatures,^28^ and has an unusually poor TGDG accumulation,^18^ we decided to focus on its activation of SFR2. We hypothesized that cotton *Gr*SFR2 would sense low temperatures differently than Arabidopsis *At*SFR2. We investigated *Gr*SFR2 activation in its native environment and heterologously in Arabidopsis and yeast in response to low temperatures, cytoplasmic acidification, and swapped protein domains. Our findings reveal a surprising divergence in activation mechanisms, enhancing our understanding of responses to low temperatures in these closely related species.

## Materials and methods

### Plant material and growth conditions

Arabidopsis (*Arabidopsis thaliana*, Columbia [Col], *sfr2* (SALK_106253), *GrSFR2, AtYFP*) were grown under two conditions. On media, they were grown as described,^7^ except the Murashige-Skoog concentration was at ½ of full strength. Soil-grown plants were grown precisely as described previously.^18^ Soil-grown plants were incubated at normal day temperatures (22°C) for 3 to 4 weeks before cold acclimation at 4°C with 12-h day/night and 60 μmol m^−2^ s^−1^ light for 1 week. Plate grown plants were incubated at normal day temperatures (22°C) with a nighttime temperature of 18°C and 120 μmol m^−2^ s^−1^ of light before cold acclimation.

*Gossypium raimondii* was grown under standard greenhouse conditions of max and min day temperature of 27°C and 24°C, respectively, and night temperatures at max 21°C and min 18.8°C. *G. raimondii* was planted with standard greenhouse soil mix [8:8:3:1 (w/w/w/w) peat moss:vermiculite:sand:screened topsoil, with 7.5:1:1:1 (w/w/w/w) Waukesha fine lime, Micromax, Aquagro, and Green Guard per 0.764 m^2^].

### Production of *Gr*SFR2 construct in Arabidopsis

*sfr2* (SALK_106253) plants were transformed using *Agrobacterium tumefaciens* (strain C58C1) carrying a construct with *Gossypium raimondii* SFR2 gene (NM_001125119.2) in pUBCYFPDest.^29^ Arabidopsis transformation was completed using the floral dip method^30^

The presence of the *Gr*SFR2 construct was confirmed by genomic PCR with forward primer 5’- GATGGTTATGGTCCCAAGTTTG-3’ and reverse primer 5’- CATGCCTGCAGGTCACTG-3’. Microscopy to confirm presence of fluorescence was done using a confocal microscope Nikon A1plus camera with a Ni-E Microscope confocal system at the Nebraska Morrison Microscopy Center with excitation at 640 nm and emission from 663 to 738 nm for chloroplast autofluorescence and 488 nm for YFP fluorescence of target protein, *Gr*SFR2-YFP.

### Arabidopsis whole plant freezing test

All plants roughly 4 weeks of age used in the freezing test were acclimated under cold conditions (4°C) under the 12-h/2-h-dark light conditions 60 μmol m^−2^ s^−1^ for 1 week prior to freezing. The freezing assay was completed as described in,^7^ altered method of.^15^ Briefly, plants were moved into a freezer at −2°C and held at this temperature for 2 hours. The temperature was then dropped to −6°C and nucleation was induced with ice chips. The plants were held at −6°C for 16 hours.

For recovery and damage assay the frozen plates were gradually warmed to room temperature for 24 hours before returning to the growth chamber prior to assessment. The light cycle for growing and cold acclimation stages followed.^31^ Recovered levels were classified and quantified by appearances. 1: fully green rosettes with minimal to no damage, the plants fully recovered, 2: partially green rosettes with partial damage, the plants partially recovered, and 3: fully white rosettes with severe damage, the plants were not able to recover. The percentage of each level within the same genotype was calculated from the sum of three biological replicates, and the total N of Col-2 = 59, *sfr2* = 49, *At*SFR2-YFP = 55, and *Gr*SFR2 = 51. The equation for recovery percentage could be expressed as below:

$$\%\mathrm{Recovery}=\frac{\mathrm{totalnumberofrosettesateachlevel}}{\mathrm{totalnumberofeachgenotype}}\times 100$$

### Cotton freezing test

Freezing was completed using a refrigerated circulator (AP15R–40, VWR, Radnor, PA, USA) and was set to first cool at a rate of −0.02°C/min to −4°C, then finally cool at a rate of −0.4°C/min to the final holding temperature of −10°C. Three leaf discs (8 mm) of cotton were immediately subjected to lipid extraction at room temperature. In tandem three leaf discs (8 mm) of cotton were placed into a tube with 1 mL water then placed into the circulator set to 0°C. After 30 minutes in the chiller, ice was added to each tube to initiate freezing. The tubes were held at −10°C overnight. The next day the tubes were left to thaw for 30 minutes at room temperature. Following this leaf tissue underwent lipid extraction described below.

### Exogenous cytosolic acidification

Arabidopsis cytosolic acidification was completed on excised leaves as described in.^18^ Cotton cytosolic acidification was completed on young leaves of vegetative-stage *Gossypium raimondii* with three or more fully expanded leaves was used for the TGDG accumulation tests. The acid test was completed directly on a fully expanded leaf by using plastic wrap with 20 mM acetic acid at pH 5.7. The acid was put in the plastic wrap and maneuvered to be on the abaxial (bottom) side of the leaf for 3 hours. During the incubation, the leaf was supported from beneath to avoid damaging the leaf or plant. After 3 hours, 6 leaf punches were taken using an 8 mm punch in the greenhouse and lipids were extracted using methods described in.^32^ All leaves were blotted dry before lipid extraction. A second excised leaf method was completed for cotton by using a 0.5 cm diameter hole punch from expanded leaves, making sure to avoid vasculature. Three discs per sample were used per assay in 20 mM pH 5 Acetic acid for either 1 hour or 3 hours. In tandem with this, each had a water control that occurred in the same manner with lipid extraction following immediately after.

### Lipid analyses

Plant lipids were extracted from the tissues using a modified Bligh and Dyer method^32,33^ and thin-layer chromatography (TLC) as described in (Z.^34^ At the end of the freezing assay described in “Arabidopsis Whole Plant Freezing Test” above, whole rosettes were sampled using forceps and tubes prechilled in liquid nitrogen prior to plant handling to minimize thawing. For leaves and punches incubated in 20 mM acetic acid, the tissue was blotted dry, gently with a paper towel prior to extraction. Lipids were extracted and stored under N_2_ gas at −80°C until use.

Yeast lipid extraction was done essentially using the modified Bligh and Dyer method^32^ except 0.1 mm diameter silicon carbide (BioSpec) and 0.5 mm diameter zirconia/silica yeast disruption beads (RPI), were used to lyse the cells in the extraction buffer. Samples were stored in amber vials under N2 gas at −80°C until processing.

Lipids were loaded onto Silica 60 thin-layer chromatography plates 1 cm from the edge and resolved in a solvent system of chloroform:methanol:acetic acid:water (85:20:10:4, v/v/v/v) as described in.^7^ Sugar-containing lipids were visualized using α-naphthol spray (2.4% α-naphthol, 80% ethanol, 10% sulfuric acid) followed by baking at 120°C (Z.^34^

### Electrolyte leakage

Electrolyte leakage was completed on Arabidopsis plants using lines, *GrSFR2, sfr2* (SALK_106253), and Col-2 as described in.^18^ The plants were grown as described above and allowed to cold acclimate at 4°C for 1 week. The fully expanded rosette leaves of Arabidopsis were used for this analysis. The leaves were put into 5 mL tubes with 3 mL of ddH2O (18 MΩ). Stepwise freezing was done using refrigerated circulator (AP15R–40, VWR, Randor, PA, USA). Conditions for Arabidopsis were determined by.^35^ The samples were allowed to equilibrate at 0°C for 30 minutes and then nucleated with a ddH2O chip at −1°C for 1 h. The stepwise chilling was then initiated and occurred at decreasing 2°C/h. Samples were collected at each time point for Arabidopsis.

After the above sampling, the leaves were left to slowly thaw at 4°C overnight. Samples were then raised to room temperature (22°C) and subsequently shaken at 250 RPM for 15 minutes.^35^ After this, initial conductivity measurement was taken using Accumet AB200 (Fisher Scientific, Hampton, NH, USA). Following this initial reading, samples were heated to 65°C for 30 minutes in a water bath to completely release all electrolytes. Leaves were then cooled to room temperature, then shaken at 250 RPM for 15 minutes. Conductivity was again measured and logged as the final leakage. For each temperature, a leaf was also sampled for lipid analysis in tandem with ion leakage.

Data for cellular leakage were analyzed as in,^35^ percent leakage relative to total ions was fit to a sigmoidal curve.

### Immunoblot analyses

Three leaves from the center of rosette of 4-week-old Arabidopsis plants were ground in liquid nitrogen, homogenized in lysis buffer (10 mM HEPES, 150 mM NaCl, 0.5 mM EDTA, 1% DDM, 1% MS-SAFE Protease and Phosphatase Inhibitor [Sigma]). The supernatant was collected after centrifugation at 20,000 × *g* for 10 min at 4°C. Equal amounts of protein (20 µg) were denatured in Laemmli buffer held at 100°C for 5 min then separated on 7.5% SDS-PAGE and transferred to PVDF membranes (Bio-Rad). Equal protein loading was confirmed by Ponceau stain. The membranes were blocked in EveryBlot Blocking Buffer (Bio-Rad) and then incubated at room temperature overnight with 1:250 anti-SFR2 antibody then washed in TBST (20 mM Tris·HCl, pH 7.5, 150 mM NaCl, 0.05% [v/v] Tween 20)

For yeast protein immunoblotting, 10 ug of protein extracts were mixed 1:1 with 2X Laemmli buffer and loaded into a 10% precast polyacrylamide gel. Proteins were resolved and then transferred to PVDF and blocked with TBST containing 5% milk powder (Carnation). Membranes were incubated with 1° anti-SFR2 (1:250)^16^ overnight and then washed with TBST.

For signal detection, membranes were incubated with 2° anti-Rabbit-HRP (1:20,000) (Invitrogen). Clarity ECL (Bio-Rad) was used to induce chemiluminescence and membranes were imaged with an Odyssey Fc (Licor).

### Plasmid generation

The CDS Cotton SFR2 (*Gr*SFR2) previously subcloned into pUC57-Kan, was used as a template for sequence swapping with regions of the Arabidopsis SFR2 (*At*SFR2) CDS. An unstructured loop, and 30 amino acid sequence close to the C-terminus in Arabidopsis SFR2 were swapped with *Gr*SFR2 sequences in this region. DNA encoding H93-H164 in *Gr*SFR2 was replaced with the DNA for S90-Lys136 from *At*SFR2 to generate the *Gr*SFR2-Loop construct. DNA encoding *Gr*SFR2 A579-L609 was replaced with the DNA for A550-L580 from *At*SFR2 to generate the GrSFR2–550/80 construct. Both constructs were commercially synthesized in pUC57-Kan (GenScript). For expression in yeast, constructs were inserted into pYesDest52 using Gateway LR Cloning (Invitrogen).

### Heterologous expression

*Gr*SFR2-Loop and *Gr*SFR2–550/80 in pYESDest52-Ura were each transformed into InvSc1 competent yeast (Invitrogen) containing CsMGD1 (pESC-His) and plated on SC-his/-ura media followed by culturing in liquid media as described in.^16^ Protein expression was induced with galactose for 8 hours, and cell pellets were either used immediately for protein and lipid extraction or stored in −80°C until use.

## Results

### *Gr*SFR2 is activated in response to freezing, but not to acidification

**Figure 1.**
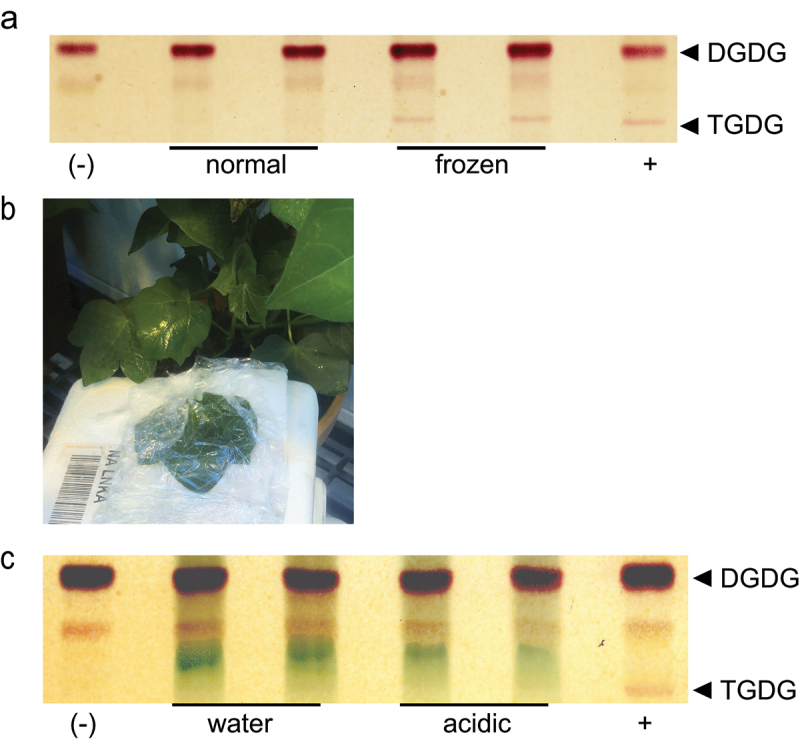
TGDG accumulation of *G.* *raimondii* during acidification and freezing (a) Thin-layer chromatogram stained for sugars and showing a separation of lipid headgroups extracted from leaf punches of *G.* *raimondii* after normal growth or freezing. Locations of digalactosyldiacylgycerol (DGDG) and trigalactosyldiacylglycerol (TGDG) are indicated at right. (b) Image of in planta cotton incubation in 20 mM acetic acid adjusted to pH 5 (c) Thin-layer chromatogram stained for sugars and showing a separation of lipid headgroups extracted after in planta leaf incubation in water or artificially acidified (acidic) conditions shown in B. Locations of DGDG and TGDG are indicated at right. Negative and positive controls represent lipid extracts of Arabidopsis leaves during normal growth (negative) or freezing (positive) conditions.

In Arabidopsis SFR2 protein is present, but not always active.^7,36^ In response to severely low temperatures, SFR2 catalyzes the production of, and subsequently causes accumulation of trigalactosyldiacylglycerol (TGDG). This phenomenon is seen in multiple species but not all, and recently cotton (*Gossypium raimondii)* was described recently to have no detectible TGDG in response to cold.^18^ To confirm if SFR2 activation does occur during freezing in *G. raimondii* leaves were excised, punched, then frozen at −10°C overnight. When treated in this manner during this assay, TGDG accumulated at very low rates during freezing, confirming that the SFR2 was activated during this freezing stress (Figure 1a). We concluded that the cotton SFR2 can be activated though to a lesser extent than previously reported for the model species Arabidopsis.^7^

In Arabidopsis, SFR2 activates when a decrease in pH occurs both internally at a cytoplasmic level or from external stimuli.^7^ It has been described that some phylogenetic groups in the angiosperms have strong differences in TGDG accumulation in response to freezing and acidic stimulation.^18^ To determine if SFR2 activation and subsequent TGDG accumulation could be mimicked in cotton, the leaves were treated with 20 mM acetic acid, pH 5 (Figure 1b,c). First, to minimize possible SFR2 activation in response to wounding of the leaf in cotton the acetic acid was held against the attached leaf and left in place with plastic wrap for 3 hours, then leaf punches were sampled for lipid extraction (Figure 1b,c). This method resulted in no TGDG accumulation within the cotton plant. To compare this method to the assay utilized in^18^ excised tissue leaf discs were put in the 20 mM acetic acid, pH 5 for 1 and 3 hours, followed by lipid extraction. TGDG was not accumulated in either method in response to external acidification unlike Arabidopsis.^7^

### Cotton SFR2 does not complement the function of *At*SFR2 in the *sfr2* mutant

**Figure 2.**
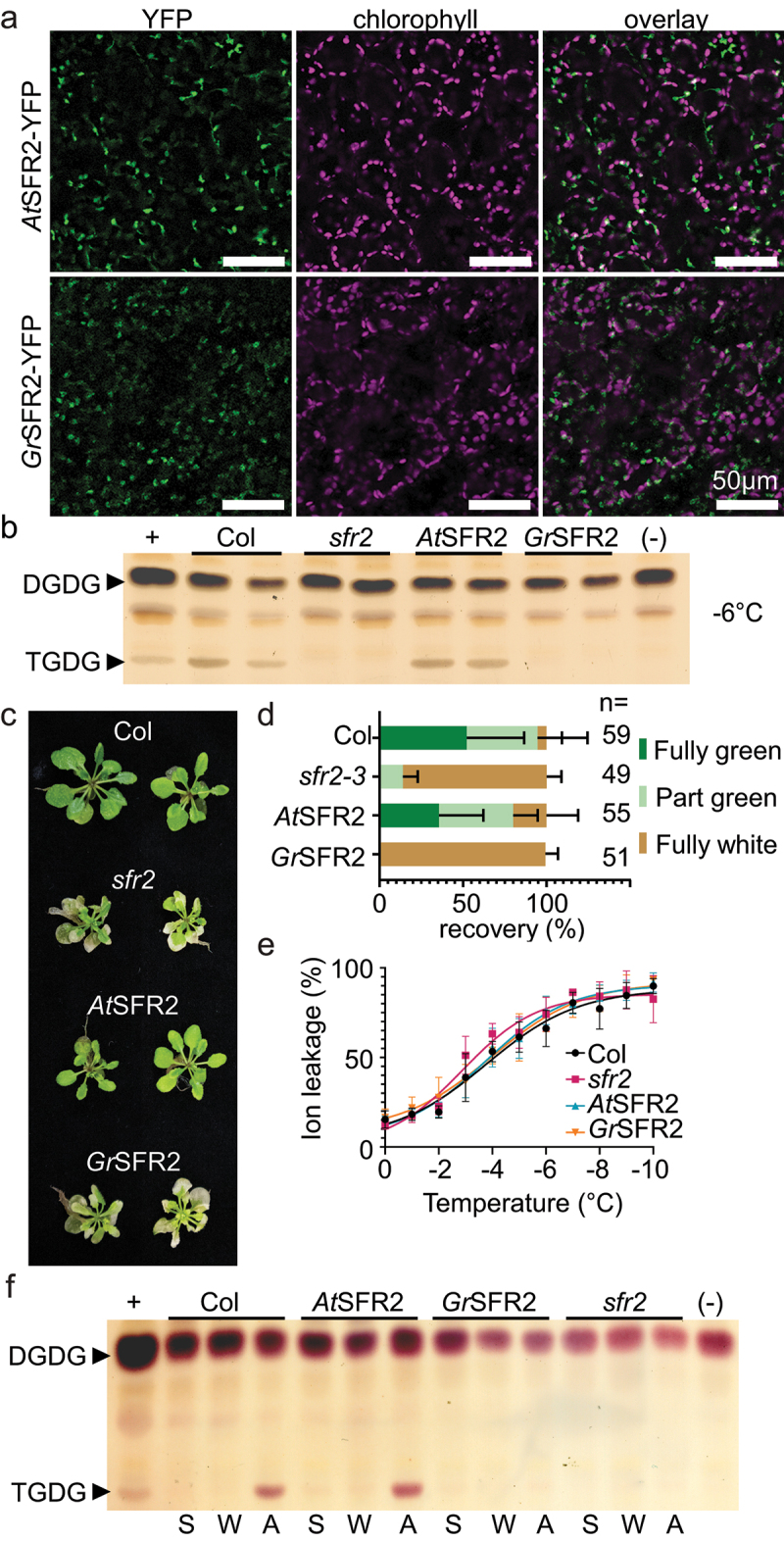
Presence, activation, and impact of *Gr*SFR2 in Arabidopsis. (a) Confocal micrographs of YFP signal, chloroplast autofluorescence, or an overlay of both signals from Arabidopsis leaf tissue expressing *Gr*SFR2-YFP or *At*SFR2-YFP as indicated at left. (b) Thin-layer chromatogram stained for sugars and showing a separation of leaf lipid headgroups from Arabidopsis genotypes indicated at the top, grown at 22°C, cold-acclimated for one week at 4°C, then frozen at − 6°C overnight, as indicated at right. Arabidopsis genotypes include wildtype (Col), SFR2 loss of function line (*sfr2–3*), *sfr2–3* expressing *At*SFR2-YFP (*At*SFR2), and *sfr2–3* expressing *Gr*SFR2-YFP (*Gr*SFR2). The locations of digalactosyldiacylglycerol (DGDG) and trigalactosyldiacylglycerol (TGDG) are indicated at left. (c) Growth phenotypes of Col, *sfr2–3*, *At*SFR2, and *Gr*SFR2 after one week of cold acclimation, overnight freezing at − 6°C, and two days of return to normal growth conditions. Phenotypes of *sfr2* and *Gr*SFR2 are similar in their inability to recover from freezing. (d) Quantification of recovery of plants treated as in panel C. Plants were manually scored for damage where “fully green” indicated no observable damage, “part green” indicated visible damage and visible growth recovery, and “fully white” indicated no visible growth recovery. Numbers of plants quantified in three growth trials are indicated at right. (e) Ion leakage from detached rosette leaves of Arabidopsis of indicated genotypes during a stepwise freezing assay from 0 to − 10°C. Data are shown as means (± SE) of 10 independent experiments. (f) Thin-layer chromatogram stained for sugars and showing a separation of leaf lipid headgroups from Arabidopsis genotypes indicated at top, after treatments indicated below. Locations of DGDG and TGDG are indicated at left. S, starting, W, treated with water, A, artificially acidified. Negative and positive controls represent lipid extracts of Arabidopsis leaves during normal growth (negative) or freezing (positive) conditions.

To inquire if *Gr*SFR2 would complement *At*SFR2, *Gr*SFR2 was transformed into an Arabidopsis mutant lacking SFR2 expression (*sfr2–3* (SALK_106253)). *In planta*, presence was visualized using YFP fluorescent tags on the *Gr*SFR2 to confirm *Gr*SFR2 presence at the known location of the *At*SFR2 protein on the surface of the chloroplast (Figure 2a).^35^ TGDG accumulation was then used as a proxy to test GrSFR2 activation. To determine if the Arabidopsis would activate *Gr*SFR2 in response to freezing, TGDG was measured in normal growth conditions, cold acclimated (6°C), and frozen plants. At normal growth temperatures and after cold acclimation, there was no TGDG accumulation for any genotype, while after freezing, TGDG accumulated in the wildtype (Col-2) and *At*SFR2-YFP/*sfr2–3* controls. TGDG did not accumulate in the *Gr*SFR2/*sfr2–3* or the *sfr2–3* plants (Figure 2b).

In addition to the accumulation of TGDG, the phenotypic response to freezing was documented in Arabidopsis expressing *Gr*SFR2. After cold acclimation and overnight freezing, the *Gr*SFR2/*sfr2–3* plants strikingly resembled the *sfr2–3* mutant background in both the subtle reduction in size and showed similar leaf damage. (Figure 2c). Quantifying the phenotype by scoring leaf damage showed that the *Gr*SFR2 plants failed to recover any photosynthetically active, green tissue while the wildtype and *At*SFR2-YFP controls were over 30% fully recovered, and over 80% partially damaged, and resumed growth post freezing (Figure 2d). This result was corroborated by a highly sensitive electrolyte leakage assay, which also showed no differences in cellular death between the genotypes throughout the freezing assay (Figure 2e). It is expected that wildtype will reach 50% (LT_50_) cellular death between −4 and −6°C, we found that there was no statistical difference between the Arabidopsis genotypes analyzed here.

To test if the activation of cotton SFR2 is initiated by external acidification like Arabidopsis, we subjected Arabidopsis expressing *Gr*SFR2 to artificial acidification using pH-controlled solutions of mild organic acid.^7^ TGDG was found in the Col-2 and *At*SFR2-YFP controls after 3 hours in response to acidification as expected, but the *Gr*SFR2 did not accumulate TGDG, instead resembling the *sfr2–3* mutant (Figure 2f) supporting the finding in the native system that *Gr*SFR2 does not activate in response to acidification of whole tissue. Together, this data suggest that *Gr*SFR2 does not activate like *At*SFR2 in Arabidopsis.

### Heterologous expression confirms critical *At*SFR2 domain regions fail to complement activation in *Gr*SFR2

**Figure 3.**
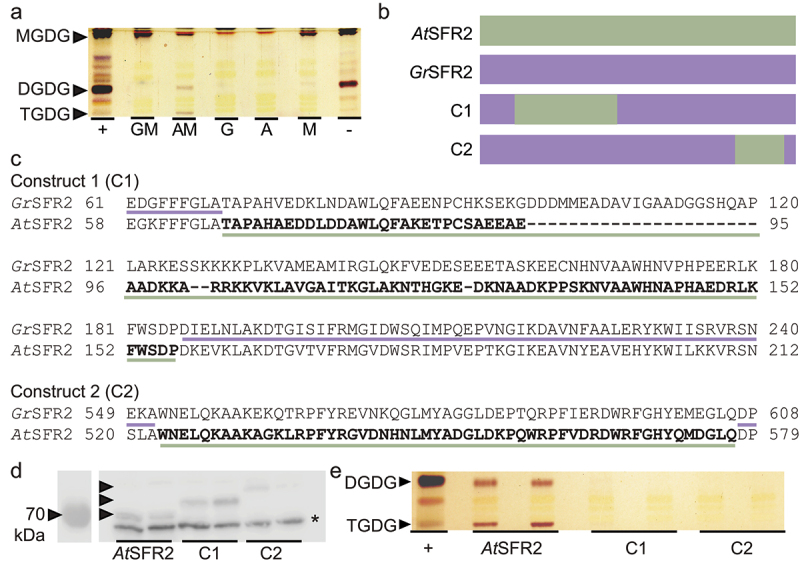
*Gr*SFR2 and *At*SFR2 region tests in yeast (pYesdest52-Ura) (a) Thin-layer chromatogram stained for sugars and showing a separation of lipid headgroups extracted from yeast expressing constructs indicated at bottom. GM is *Gr*SFR2 and monogalactosyldiacylglycerol synthase (MGD1), AM is *At*SFR2 and MGD1, G is *Gr*SFR2 alone, a is *At*SFR2 alone, M is MGD1 alone. Locations of monogalactosyldiacylglycerol (MGDG), digalactosyldiacylglycerol (DGDG), and trigalactosyldiacylglycerol (TGDG) lipids are indicated at left. (b) Depiction of yeast mutant construction, *At*SFR2, *Gr*SFR2, construct 1 (C1) made of *Gr*SFR2 with *At*SFR2 loop region, construct 2 (C2) *Gr*SFR2 with 550-580bp region from *At*SFR2. (c) Alignments showing swapped regions of *Gr*SFR2 and *At*SFR2 in C1 and C2. (d) Immunoblot detecting SFR2 loaded with equal protein (10 µg) from yeast expressing *At*SFR2, *Gr*SFR2, C1, or C2. Black arrowheads indicate SFR2 construct location and an asterisk indicates a non-specific band. (e) Thin-layer chromatogram stained for sugars and showing a separation of lipid headgroups extracted from yeast expressing *At*SFR2, C1, or C2 versions of SFR2. Locations of DGDG and TGDG are indicated at left. Negative and positive controls represent lipid extracts of Arabidopsis leaves during normal growth (negative) or freezing (positive) conditions.

We tested *Gr*SFR2 activity in a yeast heterologous expression system which shows strong activity from *At*SFR2.^16^ Yeast complemented with and without MGDG synthase and either *Gr*SFR2 or *At*SFR2 showed that when MGDG synthase is present, *Gr*SFR2 does not produce TGDG in this system (Figure 3a).

Given that *Gr*SFR2 activated differently than *At*SFR2 in both Arabidopsis and yeast systems, we speculated that sequence-based differences between the two proteins may be responsible for the difference in their activities. *At*SFR2 has two regions that are required for galactosyltransferase activity.^16^ The regions of interest from the Arabidopsis sequence are the “A loop” region located near the N-terminus region of the protein between residues 56–536 and the C-terminal region, residues 550–580 (Figure 3b and c). To investigate if these same regions could activate the GrSFR2 protein, we swapped those regions from *At*SFR2 into *Gr*SFR2, and expressed the resulting chimeras in yeast (pUC57-Kan) that also expressed MGDG synthase, allowing for SFR2 activity. The expression of the chimeric proteins was tested by immunoblotting (Figure 3d). Neither the chimeric *Gr*SFR2 with *At*SFR2 loop region, nor the *At*SFR2 550/580 region activated or accumulated TGDG differently than the original *Gr*SFR2 (Figure 3a). Thus, suggesting that the activation of cotton SFR2 is dependent on more than these domains or may differ from Arabidopsis in other regions.

## Discussion

Cotton is a cold-sensitive, economically important agricultural crop, especially to the Southeastern United States. We previously found that cotton produced undetectable levels of cold-stress-specific lipid TGDG in a large-scale screen,^18^ implying that cotton may respond to cold stress differently than model species Arabidopsis. Here we confirmed that cotton produced low levels of TGDG in response to cold (Figure 1), presumably because it retains a functional homolog of SFR2. However, *Gr*SFR2 did not respond to leaf acidification (Figure 1). When we heterologously expressed *Gr*SFR2 in Arabidopsis, it still did not activate similarly to *At*SFR2 (Figure 2). When we swapped domains of Arabidopsis SFR2 known to be critical for function into the *Gr* SFR2, *Gr*SFR2 activation remained different from Arabidopsis (Figure 3). We conclude by hypothesizing that between cotton and Arabidopsis, there has been functional divergence large enough to optimize SFR2’s stress response in each species. We note that the amount of functional divergence may be more extreme between the SFR2 homologs causing a loss of its original function. We consider the less likely of the two hypotheses because SFR2 is solely responsible for TGDG production in Arabidopsis,^15^ and cotton produces low levels of TGDG in the cold (Figure 1a), implying that *Gr*SFR2 retains function.

Stress responsive enzymes, specifically other cold responsive genes like *COR15*,^37^ *Wcs19*,^38^ and *CBF/DREB1* (W.^39^ are able to confer cold tolerance when transferred between species. Surprisingly, here when we transferred *Gr*SFR2 into Arabidopsis we were unable to recover SFR2 activity in the cold (Figure 2). Arabidopsis SFR2 is activated by acidification, and in both the native cotton system and when heterologously expressed in Arabidopsis, *Gr*SFR2 failed to activate in response to external acidification (Figures 1 and 2) further supporting the notion that cotton SFR2 is sensed and activated by different cues than those currently understood in other species.

The galactosyl hydrolase family 1 enzyme, SFR2, remodels membranes in response to a cold stress.^15,16^ Domain swapping is a common method used to determine protein functionality, for example, SYMRK proteins role in root nodule symbiosis (H.^40^ and in Cf4/Cf9 proteins to discover sequences necessary for function.^41^ Specifically, here we followed a similar approach as Li and colleagues to test the function of species-specific SFR2 proteins. In the yeast expression system, activating regions of *At*SFR2 were swapped for those of *Gr*SFR2^16^ Interestingly, *Gr*SFR2 chimeras with *At*SFR2 activation regions failed to cause activation in *Gr*SFR2 (Figure 3). This suggests that other regions of SFR2 are also needed for activation.

SFR2 is conserved across plant phylogenetic hierarchy^14^ but the accumulation of TGDG is not ubiquitous.^18^ These activation differences of SFR2 in asterids and rosids in eudicots, *and* resurrection plant have been demonstrated. Between Arabidopsis and tomato specifically, tomato SFR2 activity was nearly twice that of Arabidopsis under the same conditions.^42^ In *Craterostigma plantagineum*, a resurrection plant, *SFR2* transcript is upregulated and TGDG levels increase in response to dehydration.^43^ Our findings corroborate that despite the close evolutionary relationship of the species and sequence similarity, an enzyme’s activity can vary greatly and depend on different environmental cues. These findings suggest that at least some membrane stress responses can be tuned within a short evolutionary timescale toward different stresses, as Arabidopsis SFR2 responds primarily to low temperature, tomato to high salt, and *C. plantagineum to* desiccation. Our study extends this observation to conclude that the molecular mechanisms of signaling differ in cotton than prior studies in other species (acidification did not activate *Gr*SFR2, Figures 1 and 2, as do the mechanisms of sensing the signal (*Gr*SFR2 chimeras could not sense *At*SFR2 environment). This raises the question of how best to engineer similar traits to improve crop cold tolerance. Discovering how to improve the cold tolerance of cotton is important for continued improvement to its agricultural production.

## Data Availability

No large datasets are associated with this work. Raw image files supporting plant growth and chromatography conclusions are available upon request.

## Cover image

Generated by Adobe Firefly AI https://firefly.adobe.com/generate/images.
